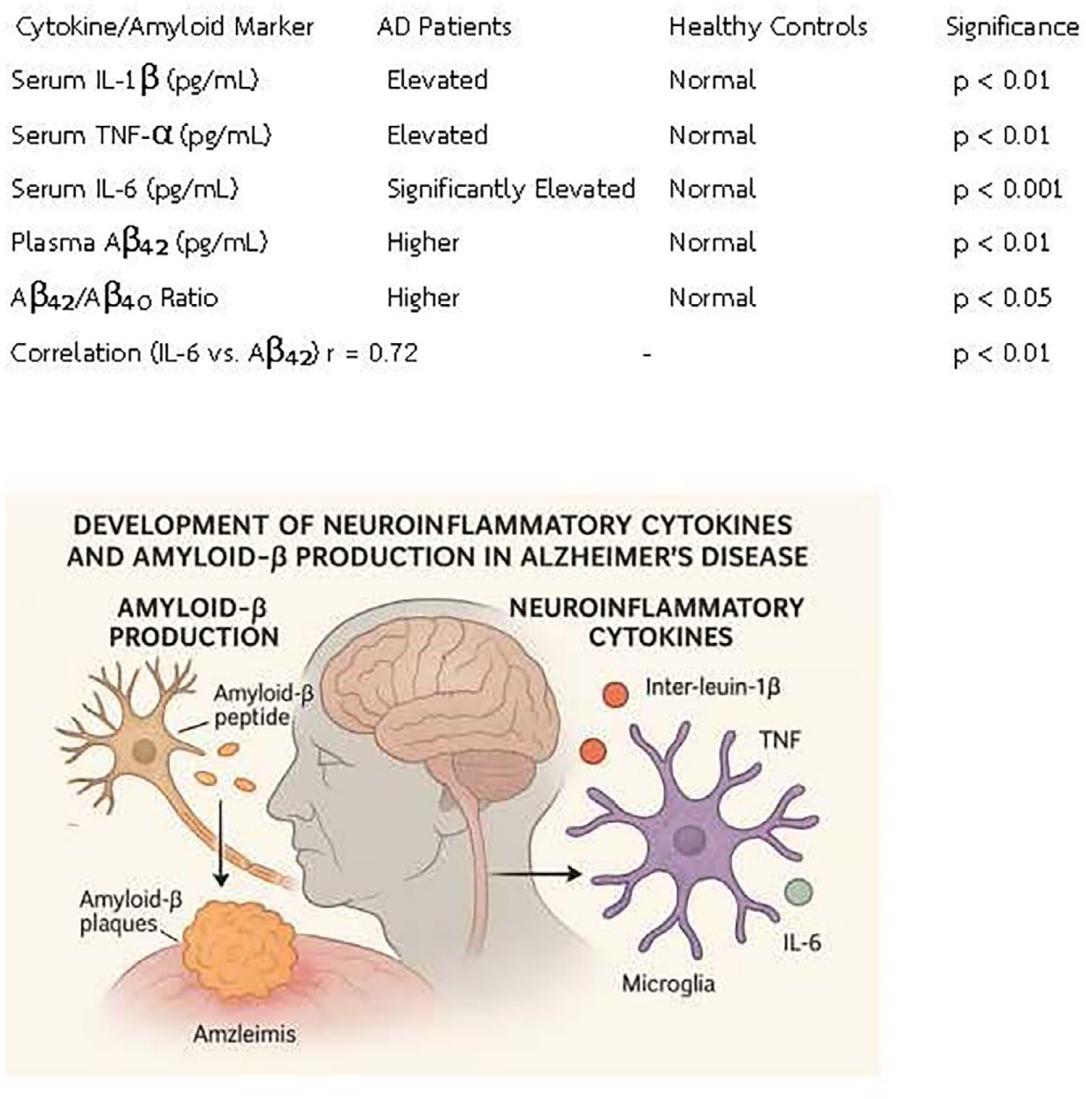# Development of Neuroinflammatory Cytokines and Amyloid‐β Production in Alzheimer's Disease

**DOI:** 10.1002/alz70861_108283

**Published:** 2025-12-23

**Authors:** Chanikarn Sonpee, Chanida Ruchisrisarod, Watayuth Luechaipanit, Pasin Hemachudha, Thirawat Supharatpariyakorn, Abhinbhen Wasontiwong Saraya

**Affiliations:** ^1^ Kingchulalongkorn Memorial Hospital, Pathumwan, Bangkok Thailand; ^2^ Thai Red Cross Emerging Infectious Diseases Health Science Centre, King Chulalongkorn Memorial Hospital, Bangkok Thailand; ^3^ Faculty of Medicine, Chulalongkorn University, Bangkok Thailand; ^4^ Chula Neuroscience Centre, Bangkok, Bangkok Thailand; ^5^ Division of Neurology, Department of Medicine, Faculty of Medicine, Chulalongkorn University, Bangkok Thailand

## Abstract

**Background:**

Alzheimer’s disease (AD) is a chronic and progressive neurodegenerative disorder characterized by memory loss, cognitive decline, and behavioral impairment. A central hallmark of AD pathology is the abnormal accumulation of amyloid‐β (Aβ) plaques and neurofibrillary tangles, accompanied by a sustained neuroinflammatory response. Emerging evidence suggests that inflammatory mediators, both central and peripheral, play a significant role in the pathogenesis and progression of AD, particularly neuroinflammatory cytokines, which influence Aβ production, aggregation, and clearance.

**Methods:**

This study aimed to explore the association between systemic inflammatory cytokines and circulating Aβ levels in the peripheral blood of AD patients. Serum levels of pro‐inflammatory cytokines, including interleukin‐1β (IL‐1β), tumor necrosis factor‐α (TNF‐α), and interleukin‐6 (IL‐6), were measured in AD patients and compared with age‐matched healthy controls. Additionally, plasma Aβ₄₂ levels and the Aβ₄₂/Aβ₄₀ ratio were assessed to investigate amyloid metabolism in the periphery. Correlation analysis was performed to examine the relationship between cytokine levels and Aβ concentrations.

**Results:**

AD patients exhibited significantly elevated serum levels of IL‐1β, TNF‐α, and IL‐6, with IL‐6 showing the most pronounced increase. Plasma Aβ₄₂ levels and the Aβ₄₂/Aβ₄₀ ratio were also significantly higher in AD patients, indicating altered amyloid metabolism in the peripheral circulation. A strong positive correlation was observed between IL‐6 levels and plasma Aβ₄₂ concentrations (r = 0.72, *p* < 0.01), suggesting that systemic inflammation may contribute to amyloid dysregulation in AD.

**Conclusion:**

These findings support the potential use of blood‐based biomarkers for the early detection and monitoring of AD. The study highlights the significance of targeting neuroinflammatory pathways as a potential therapeutic strategy to mitigate Aβ accumulation and slow disease progression. A deeper understanding of the molecular interactions between inflammatory cytokines and Aβ could open new therapeutic avenues for AD intervention.